# Beyond Food Promotion: A Systematic Review on the Influence of the Food Industry on Obesity-Related Dietary Behaviour among Children

**DOI:** 10.3390/nu7105414

**Published:** 2015-10-16

**Authors:** Diana Sonntag, Sarah Schneider, Noreen Mdege, Shehzad Ali, Burkhard Schmidt

**Affiliations:** 1 Mannheim Institute of Public Health, Social and Preventive Medicine, Medical Faculty Mannheim of the Heidelberg University, Mannheim 68167, Germany; sarah.schneider31@gmx.de (S.S.); burkhard.schmidt@medma.uni-heidelberg.de (B.S.); 2 Department of Health Sciences, University of York, York YO10 5DD, UK; noreen.mdege@york.ac.uk (N.M.); shehzad.ali@york.ac.uk (S.A.)

**Keywords:** obesity, childhood, food industry, fast food, soft drinks

## Abstract

An increased consumption of energy-dense, nutrient-poor food and beverages as a result of a changing obesogenic environment contributes substantially to the increasing prevalence of childhood overweight and obesity. This paper reviews the nature and extent of food industry influences which expose children to commercial influences and thus might affect unhealthy dietary behaviour and finally contributes to obesity. A systematic search of nine electronic databases (including PubMed, PsycINFO, EconLit) and reference lists of original studies and reviews using key search terms identified 1900 articles. Of these only thirty-six articles met the inclusion and quality criteria. A narrative synthesis of the reviewed studies revealed six key obesogenic environments by which the food industry possibly influences obesity-related dietary behaviours in young children. These were schools, retailers, mass media “television”, mass media “internet”, home and promotional campaigns. Identifying these obesogenic environments is critical for monitoring and controlling the food industry, the development of effective environmental-level interventions to prevent childhood overweight and obesity and to identify knowledge gaps to be addressed in future research to support informed decisions of policy makers.

## 1. Introduction

The increasing prevalence of childhood overweight and obesity is receiving significant public, political and economic attention. The primary reason is that childhood BMI is associated with significant long-term health and economic consequences [1]. There are several factors that have been identified in the literature as potential determinants of the growth of childhood overweight and obesity. Among the key contributors are “obesogenic environments”, defined by Swinburn and colleagues as “the sum of the influences that the surroundings, opportunities or conditions of life have on promoting obesity” [2,3]. In their “obesogenic environmental framework” Swinburn and colleagues [3] highlight that individuals interact with multiple micro environments or local settings such as schools, homes and food retailers, that involve food, physical activity or both. The obesogenic micro environments can contribute to overweight and obesity by encouraging unhealthy diets in terms of increased consumption of energy-dense, nutrient-poor food and beverages; and inadequate exercise, for instance due to changes in leisure activities [2,4]. The food and physical activity within micro environments are in turn influenced by broader macro environments and actors such as health systems, the food industry and government policies, which are often beyond the control of individuals.

A number of systematic reviews have analyzed the nature and extent of ubiquitous food promotion [5,6,7,8]. As one of the many components of the macro environment, food promotions influence children’s preferences, consumption and purchasing requests to parents. Most studies included in these reviews focus on documenting the effects of television-based food promotion on children, with the main finding being that the majority of such marketing is for unhealthy foods [5]. Relatively few studies provide evidence for further environmental influences on children such as parents who buy unhealthy food or classmates who consume unhealthy food or beverages [9,10]. In addition, only a few reviews have attempted to evaluate the complexity (nature and extent) of obesogenic environments [11,12,13] and how environmental determinants influence eating behaviours [14,15]. One of these reviews focused primarily on the child population to examine environmental influences on dietary determinants of childhood obesity such as food promotion, availability and access [13]. However, this review did not comprehensively address all avenues e.g., internet-based advertising through which the food industry influences children’s dietary behaviour. In addition, the search strategy in the study was narrow and only focused on nine components of food environment derived from a stakeholder workshop and quantitative studies, and excluded studies focusing on non-food influences.

The debate on the influence of the food industry on eating behaviour and childhood obesity has been ongoing. Both micro and macro environments are becoming more diverse and the evidence on mechanisms and level of influence of the food industry is growing. New avenues of food promotion, such as internet-based advertising, mobile phone apps and games, have come to the forefront of the debate. These developments require a detailed reassessment of the evidence using a comprehensive conceptual framework. Sonntag and Schneider developed a conceptual model that draws on the obesogenic environmental framework [3], and identifies five obesogenic macro environmental and three micro environmental components through which the food industry potentially influences dietary behaviours in young children (see, supplementary 1) [16]. Based on this conceptual model, the primary aim of this review is to identify the nature and extent of food industry influences and how they might affect unhealthy dietary behaviour. A secondary objective is to detect overlapping patterns across these environments and how these activities might affect children’s attitudes towards food, intake patterns and their weight.

## 2. Experimental Section

### 2.1. Methods

Due to the novelty, complexity and broadness of the proposed research question, the review was designed *a priori* utilizing the Assessing the Methodological Quality of Systematic Reviews (AMSTAR) criteria as guidance, which specifically addresses comprehensive literature searches, to ensure methodological quality [17]. The systematic review followed the principles recommended by the Centre for Reviews and Dissemination guidance for undertaking systematic reviews [18]. The reporting procedures followed the Preferred Reporting Items for Systematic Reviews and Meta-Analysis (PRISMA) guidance [19].

### 2.2. Search Strategy

A search strategy was developed in cooperation with a Cochrane expert from the University Library of Heidelberg. The strategy aimed to identify all published studies that analyze the nature and the effects of dietary-related environments influenced by the food industry on young children. The search terms were developed drawing on the conceptual model that identifies five obesogenic macro environmental and three micro environmental components through which the food industry potentially influences dietary behaviours in young children (see supplementary 1) [12]. The search terms are related to the target population, child-related environments and outcomes that are influenced by the food industry. The following electronic databases were searched to identify relevant studies from inception to March 2014: PubMed, Web of Science Core Collection, The Cochrane Library, PsycINFO, PSYNDEX, EconLit, Business Source Premier, WISO Wissenschaften and Medpilot (see supplementary 2). The reference lists of retrieved articles were also searched to identify potentially relevant studies.

#### 2.2.1. Inclusion Criteria

Studies were included if they: (i) were conducted in European countries, USA, Canada, Australia or New Zealand since obesity epidemics differ between developed and developing countries; (ii) enrolled children aged from three to 11 years since there is strong evidence that mass media influence the food and beverage preferences of children ages two to 11 years [8]; (iii) evaluated food industry marketing strategies on various dietary-related environments of young children and (iv) were published in a peer-reviewed journal in English, German or French since most relevant studies were published in these three languages in the past. Studies that analyzed the effects of programs implemented by governments, non-governmental organizations or schools on dietary behaviours in children to focus on and isolate primarily the potential influence of the food industry were excluded. Studies that provided insufficient empirical evidence for our research question, or were rated with a poor or fair quality score were also excluded.

#### 2.2.2. Study Selection and Data Extraction Strategy

Two reviewers independently screened all study titles and abstracts for inclusion. Full articles of the potentially relevant studies identified from this first stage were retrieved and independently screened for eligibility by two reviewers. Disagreements were resolved through discussion, and the reasons for exclusion were recorded for each of the excluded full text articles. We calculated the inter-rater reliability for abstracts and full text screening by using Cohens Kappa, which is a measure commonly used to evaluate the compliance of independent raters [20].

Two researchers also conducted data extraction independently, with disagreements being resolved through discussion. The data extracted included the target population characteristics, marketing technique, outcomes (e.g., effectiveness outcomes), environments (school, home, internet and TV *etc.*), methodological characteristics (study design (e.g., quantitative *versus* qualitative)), as well as detailed study background information (e.g., authors, year of publication *etc.*).

#### 2.2.3. Quality Assessment

A quality assessment tool that is specifically developed for applicability to any type of research method [10] was adapted since the review was most likely to include studies utilizing a diverse range of research methods (e.g., qualitative and quantitative; descriptive, experimental and quasi-experimental designs). Five items were assessed: (1) Does this study address a clearly focused research question? (2) Was a theoretical model/framework used? (3) Is the methodology used in the study appropriate for the research question? (4) Are relevant limitations acknowledged? (5) Are the main findings clearly stated, including a summary of the strength of evidence for each main outcome? Each of the studies identified from the full text eligibility assessment was then rated on each of these criteria using three dimensions (1) not met; (2) partially met and (3) definitely met. A quality score was then calculated based on the mean score of these three quality dimensions, and studies were categorized according to the following three levels of quality: (i) poor (<60 percent); (ii) fair (60–90 percent) and (iii) good (>90 percent) [21].

### 2.3. Analysis/Synthesis of Studies

Due to substantial heterogeneity between the studies, a meta-analysis was not possible. Therefore, a narrative synthesis of the included studies was conducted to summarize the key features and compare research questions, methods and results.

## 3. Results

### 3.1. Literature Search

**Figure 1:**
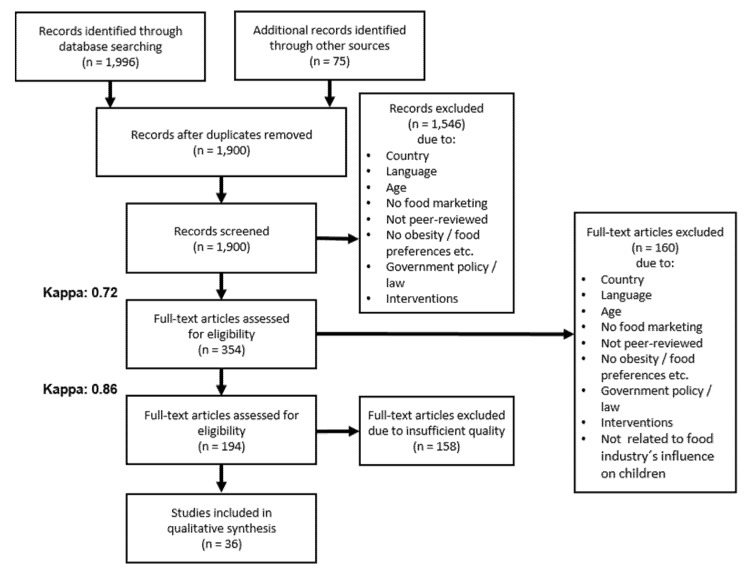
PRISMA Flow Diagram.

We identified 1,996 articles from the database searches (see supplementary 2 for detailed presentation of the search strategy), and an additional 75 articles from reference lists of the identified articles (see Figure 1). Nineteen hundred articles remained after removal of duplicates; 354 potentially relevant studies were identified from screening titles and abstracts, and retrieved as full texts. Following full text review, 160 of these studies were excluded, leaving 194 studies. While these studies addressed the influence of the food industry on diet-related environmental factors of childhood overweight and/or obesity, often research designs and methodology did not sufficiently deliver relevant empirical evidence. Only 36 out of the 194 studies were eligible and were included in the narrative synthesis. Inter-rater reliability for title and abstract screening rendered a Cohens’ Kappa of 0.72 and of 0.86 for full text selection respectively (Figure 1).

### 3.2. General Characteristics of Studies

Table S1 reports the key characteristics of included studies (see Supplementary Material). There was a wide variation among included studies in terms of target population and methodology. The majority of studies were conducted in the United States (22), followed by the United Kingdom (5), Canada (5), Australia (4), the Netherlands (4), France, Spain, Germany and Sweden (1). Whilst most studies examined a broad range of ages (about 2 to 11 years), nine had a narrow age range of two or three years [22,23,24,25,26,27,28,29,30]. With respect to methodology, four studies were descriptive [29,31,32,33], ten were content analysis [34,35,36,37,38,39,40,41,42,43], five were quasi-experimental [9,44,45,46,47], nine were experimental [22,23,27,28,30,48,49,50,51] and seven were correlational studies [24,25,26,52,53,54,55].

### 3.3. The Influence of the Food Industry on Children’s Unhealthy Dietary Behaviour and Weight

In Table S2 we grouped the studies included in our review into six obesogenic environments influenced by the food industry to alter dietary behaviours in children: school, home, media “internet”, media “television”, promotional campaigns and retailers (see Supplementary Material). Table S2 also summarizes: the marketing techniques each study examines; and whether an influence of industry was found on children’s dietary behaviours and preferences, or weight. The results are summarised below.

#### 3.3.1. School

We retrieved only one relevant study by Briefel *et al.* [31] examining the influence of food companies in schools through pouring rights contracts, food offered by brand-name restaurants and vending machines. Pouring rights contracts are contracts between an institution and a beverage maker or distributor guaranteeing the latter the exclusive rights to sell its products at the particular institution. They found that schools without snack bars or pouring rights contracts where beverage manufacturer exclusively control the beverage distribution in school showed significantly lower intake of sugar-sweetened beverages and energy-dense food per school child.

#### 3.3.2. Home

Four articles analysed whether advertisement to parents might influence diet-related behaviours in children [9,33,41,52]. While these studies did not provide evidence for a causal relationship between advertisement directed to parents and children’s weight, they identified pathways by which the food industry may adversely influence children through its effects on parents. For example, Manganello *et al.* [41] found that parenting and family magazines include a large number of food advertisments; and most of them were for highly processed foods such as snacks, cookies and fast food. Moreover, Grier *et al.* [9] examined whether such an increased parental exposure may influence children and found that higher exposure to fast food marketing for parents, especially advertising and in-store promotions, was associated with more frequent fast-food consumption by their children. Similarly, Jones and Fabrianesi, confirmed that parents are also more likely to purchase highly processed food which is advertised as nutritious, healthy, tasty and convenient [52]. By contrast, the findings of another study indicate that mothers perceive the negative influence of food advertisement (particularly on their children) and try to manage healthy eating habits within their families [33].

#### 3.3.3. Internet

Only three studies analysed the nature and effects of online food marketing on children [38,40,53]. Their findings suggest that so-called “advergames” may have a significant influence on diet-related behaviours in children. Specifically, most advergames promoted unhealthy food and did not include an age-limit specification. This raises the possibility that children may have access to advergames that are not child-age appropriate. Children are particularly susceptible if advergames include a high number of brand identifiers, or when they can play these games without any restriction [53]. Brand-related messages within a game, as one out of six features to influence children, are primarily used by for-profit advergames that intend to increase sale [38].

#### 3.3.4. Television

The majority of articles included in our review analysed the effects of television food advertisement on children. Among them seven studies provided a content analysis of food advertisements and found that particularly high fat, salt or sugar (HFSS) products such as sugary cereals and sweets, fast food, chips/crackers *etc.* were very frequently advertised in highly rated children’s cable channels. By contrast, the proportion of television advertisement for fruits, vegetables and juices were small (less than two percent) [36]. While these studies found evidence that high-energy food is predominantly advertised in children’s television programs, they only describe the nature of food advertising but not its effects on children. 

We found seven articles that examined the effects of food advertising on the following child outcomes (i) preferences; (ii) consumption and (iii) body-mass-index (BMI) [22,23,44,45,46,49,50]. Two studies analyzed the effect on children’s preferences and confirmed that their preferences for an advertised product increased after watching food commercials on television [22,49]. Dixon also reported that a combination of junk food and healthy food advertisements does not change the effects of the junk food advertisement, but has a negative influence on children’s attitudes towards vegetables [22]. While these studies demonstrated that food advertising results in increased preferences for HFSS products and requests to parents to buy these products, they did not examine effects on consumption and childhood obesity. Four studies [23,44,46,50] provided evidence that food advertising increases children’s consumption of energy-dense food; and that overweight children are particularly vulnerable to food advertising [46,50]. However, none of these studies provided direct evidence of changing BMI in the long run as a consequence of the increased consumption of advertised products. One longitudinal study [45] that analysed the effects of total television viewing on BMI found that advertisement did not significantly alter children’s BMI. Their results indicate that television advertising is only one critical factor in the relationship between television viewing and children’s weight. This suggests that restrictions on television advertising to children on their own are unlikely to have a significant effect on childhood overweight and obesity. 

Six studies included in our review analysed the effects of self-regulated restrictions on television food advertising to children [24,25,26,42,47,55]. Their results varied substantially. While three studies found evidence that self-regulation is positively correlated with the amount of food and beverage advertising [24,25,42], three studies found the opposite [26,47,55].

#### 3.3.5. Promotional Campaigns

Six articles analysed the effects of food industry promotional campaigns on children’s dietary preferences and consumption patterns [27,28,29,30,48,51]. From a young children (below age eight) are cognitively not able to recognize the real purpose of advertisement, they are highly susceptible to such an influence and in addition, they can easily remember contents of advertisements or recognize brands [27,48]. Particularly, collectible toys increase children’s brand awareness [30]. Moreover, three studies [27,29,51] found that children’s awareness of brands is higher for unhealthy than for healthy food. While Kopelmann *et al.* did not confirm a relationship between children’s higher ability to recognize brands and their consumption; Jones and Kervin as well as Forman *et al.* found that children also consumed more in the presence of food brands [27,29,51]. Similarly, Keller *et al.* confirmed the link between brand recognition and consumption and showed that particularly overweight children eat more when foods were branded [28]. None of these studies analysed whether food branding also changes the longer term eating behaviour and thus contributes to a tracking of overweight and obesity from children to adolescents. 

#### 3.3.6. Retailers

We retrieved only three studies that examined how the food environment in retailers affects children’s preferences and whether they can influence their parents’ willingness to buy unhealthy food [13,32,37]. While two studies focussed on supermarkets, one study also considered the diversity of environments around supermarkets such as proximity to children’s home [13]. Berry and McMullen [37], for example, analysed the product display in supermarkets and found that breakfast cereal products with higher-than-average levels of sugar, refined grains and trans-fats were more likely to use child-oriented features in the form of (i) child-oriented colours including red, orange and yellow (90 percent); (ii) spokes characters such as Kellogg’s Tony the Tiger (34 percent) or (iii) premium offers such as toys, games and coupons (35 percent). Since the majority of these products were accessible to children, they could influence their parents’ purchase decisions by making verbal requests for these special products. Ogba and Johnsson [32], also confirmed an influence of children’s preferences on parents’ purchase of unhealthy food.

### 3.4. Quality Assessment

**Figure 2:**
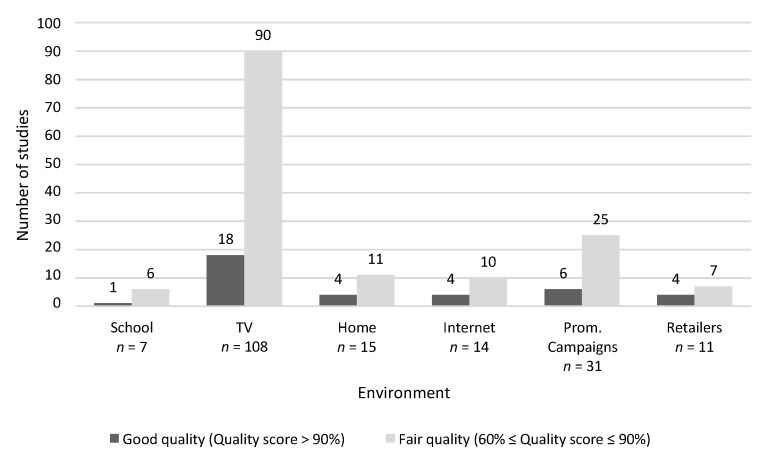
Quality Assessment—number of studies with fair and good quality (seven additional studies, which provided an overview over the influence of the food industry on children, were identified as being of fair quality and were not included in the figure. None of the studies examined more than one channel).

Quality assessment was conducted on 194 full text articles that were judged as potentially eligible after full text assessment. As already indicated above, only 36 of these were rated with a good quality score (>90 percent) (Figure 2).

Most of these 36 studies had a clear and focussed research question, and set the stage for it by summarizing the current state of knowledge. With respect to methodology, the majority of studies used qualitative tools such as questionnaires or quantitative analyses that were appropriate for the research question. This is also true for most of the studies that conducted content analyses. Since many aspects of the influence of the food industry on childhood obesity are still unexplored, more extensive content analyses are needed in order to describe the specific nutritional content of advertised food. This would allow gauging of the effects of the product itself or any potential change of food promotion. Cross-sectional studies analysed causal effects of food promotion on children. However, generalizability of findings to different settings was often limited due to the following reasons: (i) non-representative sample [24,27,49,51,53]; (ii) missing long-term effects and (iii) analysis of specific products, time periods, age-groups [23,24,25,26,44,53,54,55]. Overall, a large variety of methods were applied to measure the influence of the food industry on children’s dietary behaviours, which restricts the comparability of the results across studies. 

Only two studies [39,47] did not include any study limitations or failed to acknowledge all relevant issues. The remaining studies explained limitations in detail; particularly with respect to self-reported data and limitations to the generalizability of results to different settings and ethnical groups. While the majority of studies summarized key findings for each main outcome including the strength of evidence, three studies did not directly refer to their hypotheses and only partially examined the relationship mentioned in our research question [44,47,50].

## 4. Discussion and Conclusions

The results of this review highlight six key obesogenic environments through which the food industry influences obesity-related dietary behaviours in children: schools, retailers, mass media “television”, mass media “internet”, home and promotional campaigns. Two emerging features were identified from the evidence. First, food products that are high in sugar, fat and sodium such as fast food, sweets, snacks and unhealthy cereals are overwhelmingly promoted while healthy foods high in fiber, vitamins and minerals such as fruits and vegetables are often less advertised [36,41,42,54,56]. Second, the food industry uses persuasive marketing techniques such as attractive product packing, toys, and emotional appeals to forge long-lasting relations with children and create brand loyalty in the short and long run [27,29,30,32,37,38,39,57].

The review has also revealed a number of evidence gaps. Firstly, previous research has mainly focused on short-term effects on children’s immediate food preferences and consumption [9,22,23,27,28,29,31,32,33,44,46,49,50,52], with only a few studies analyzing long-term effects on BMI [13,45]. Secondly, most of the currently available evidence focuses on the environmental exposure “television”, with resulting research gaps in other obesogenic environments which still need to be addressed. Thirdly, most of the current evidence examines upstream determinants of childhood overweight and obesity, with only a few studies directly assessing markers of childhood overweight and obesity and reporting conflicting results [44,45]. Considering the complexity of the associations between obesogenic environments and childhood obesity, research utilizing more objective and clinically relevant outcomes could support decision making at policy level with regard to restraining food industry activities. This complex association is not unique to studies on children, but has also been demonstrated in studies on adults and adolescents [11]. 

This study focuses on the plurality of obesogenic diet-related environments through which the food industry influences children´s dietary behaviour. Our review is broader than other recent systematic reviews [5,6,7,8] that have focused on specific obesogenic environments such as television. Also, unlike Osei-Assibey *et al.* [13], our review is not limited to intervention studies or longitudinal studies and included important evidence from other study designs such as qualitative studies. The current review also uses a broader approach based on hypotheses from theoretical conceptual models [14] to address the complexity inherent to the topic. By so doing, it provides an important step forward in mapping the complexity of obesogenic environments and their influence on eating behaviour [11,13,14,15]. Finally, we only included studies that were judged to be good quality; to our knowledge, this was not done in previous reviews of (child-related) obesogenic food [11,13].

Our review also has some limitations. Firstly, publication bias (whereby positive studies are more likely to be published than negative studies) and selection bias (owing to our language limitation to only English, German and French articles) has to be taken into account. Secondly, the wide range of methods used by studies precluded the use of meta-analysis [10,13]. Thirdly, few evidence outcomes in our review were reported in anthropometric indices linked to childhood overweight and obesity. Thus, most studies assumed that a changing intermediary behaviour (such as changes in eating behaviour or nutrition knowledge) is a requirement for changes in children´s weight. Fourthly, since we explicitly restricted the search strategy to children, we excluded studies that address the same issues in adults e.g. perception and effects of curtain colours of a product. Focusing on children seems reasonable as compared to adults; children are more vulnerable to persuasive advertisement techniques, which imply that the magnitude of the food industry’s influence on dietary behaviour and long-term consequences for children are greater [48]. Finally, the conceptual framework utilized for this review does not comprehensively cover other multiple networks of further macro environmental sectors such as public transportation systems, sport/leisure industry, governments within which the food industry is embedded.

Given the consistency observed in our findings that the food industry influences children’s preferences and consumption for high-energy dense products, additional studies will have limited value except to elucidate the effects of the food industry on specific sub-populations such as low-income families that are so far only analyzed by one study [46]. Rather, the debate should now center on how this evidence can be integrated in public policy to support governments and private actors to create healthy food environments where the influence of the food industry is mitigated. Recently a couple of new broad food policy frameworks have been launched such as the International Network for Food and Obesity/Non-communicable Diseases Research, Monitoring and Action Support (INFORMAS) framework and the World Cancer Research Fund International Nourishing framework. While INFORMAS acts on the international level by providing the first coordinated global framework that tracks the characteristics of food environments, and policies and actions of public and private sectors influencing food environments [58] the Nourishing framework supports governments on the level of the food system by identifying where actions are needed to promote healthy diets and select and tailor options suitable for their populations [59] Following these pioneering frameworks, existing food policy frameworks need to be extended to include new, influential environments that are relevant to policy making. The focus of such an approach would shift the current child-centric (downstream) perspective to a socio-ecological (upstream) perspective. Such an up-stream approach is important with respect to effective public health policy and public health interventions to prevent childhood overweight and obesity. Tailored interventions targeting the six environmental exposures examined here are likely to hold promise to positively influence children’s dietary nutrition. Following the recommendations of the United Nations rapporteurs, such an upstream approach has to be accompanied by an independent controlling and monitoring of the food industry rather than self-regulation which has proven to be ineffective so far [60].

Our review summarizes an extensive body of literature on the key obesogenic environments influenced by the food industry and their effects on unhealthy dietary behaviours of children. Our findings point to the need to shift from child-centric perspective to an upstream perspective that takes into account the direct influence of key obesogenic food environments on children. Our review and the perspectives above provide a foundation for public health policy.

## Supplementary Materials

Supplementary materials can be accessed at: http://www.mdpi.com/2072-6643/7/10/5414/s1.
