# Supplementary material for: Beyond Food Promotion: A Systematic Review on the Influence of the Food Industry on Obesity-Related Dietary Behaviour among Children
Source: Nutrients. 2015 Oct 16;7(10):8565–76. doi: 10.3390/nu7105414 (PMC4632434; doi:10.3390/nu7105414)
Supplement: Supplementary file 1 [file nutrients-07-05414-s001.docx]

Supplementary 1: Impact Model


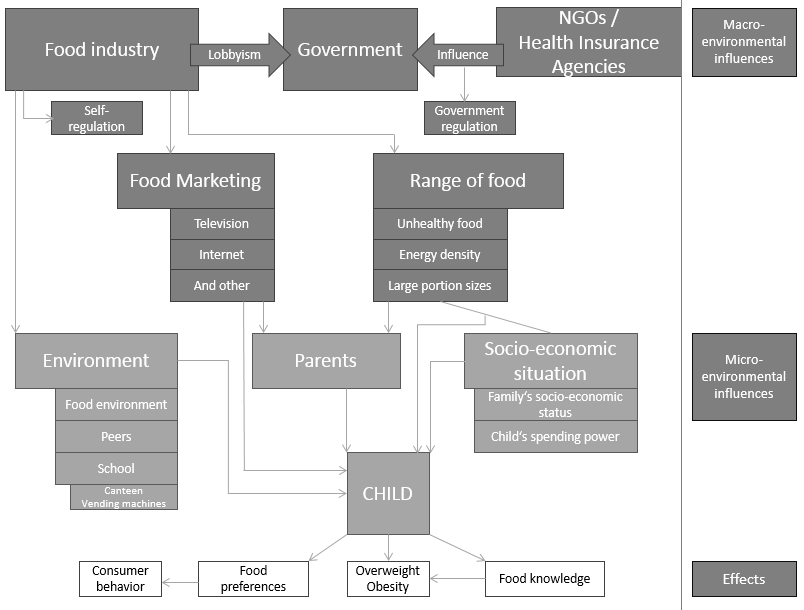


**Figure S1.** Source: Sonntag and Schneider (2015).

Supplementary 2: Search Strategy

The following electronic databases were searched in March 2014:

1. PubMed

2. Web of Science Core Collection

3. The Cochrane Library

4. PsycINFO, PSYNDEX, EconLit, Business Source Premier (via EBSCO Host)

5. WISO Wissenschaften

6. MEDPILOT

PubMed, 912 results

1. overnutrition[mh] OR overweight[mh] OR obes*[tiab] OR adipos*[tiab] OR excess weight[tiab] OR overweight[tiab] OR overnutrition[tiab] OR fatness[tiab]

2. child[mh] OR child, preschool[mh] OR child*[tiab] OR preschool*[tiab] OR pupils[tiab] OR schoolchild*[tiab]

3. health knowledge, attitudes, practice[mh] OR diet[mh:noexp] OR food and beverages[mh] OR food industry[mh] OR food habits[mh] OR child nutritional physiological phenomena[mh:noexp] OR eating[mh] OR drinking behavior[mh:noexp] OR diet[tiab] OR dietar*[tiab] OR food*[tiab] OR eat*[tiab] OR snack*[tiab] OR sweets[tiab] OR candy[tiab] OR fastfood[tiab] OR junkfood[tiab] OR beverage*[tiab] OR lemonade*[tiab] OR soda pop*[tiab] OR drink*[tiab] OR softdrink*[tiab]

4. internet[mh] OR play and playthings[mh] OR persuasive communication[mh] OR advertising as topic[mh] OR public relations[mh:noexp] OR marketing[mh] OR product packaging[mh] OR portion size[mh] OR internet[tiab] OR web*[tiab] OR app[tiab] OR apps[tiab] OR campaign*[tiab] OR vending machine*[tiab] OR automatic dispens*[tiab] OR canteen*[tiab] OR school cafeteria*[tiab] OR school tuck shop*[tiab] OR product placement*[tiab] OR advergame*[tiab] OR game*[tiab] OR toy*[tiab] OR plaything*[tiab] OR advert*[tiab] OR promotion*[tiab] OR “public relations”[tiab] OR marketing[tiab] OR sponsor*[tiab] OR brand*[tiab] OR commercials[tiab] OR packag*[tiab] OR “product design”[tiab] OR portion size*[tiab] OR large portion*[tiab] OR larger portion*[tiab] OR large food portion*[tiab] OR larger food portion*[tiab] OR “in store”[tiab]

5. health promotion[mh] OR health promotion*[tiab]

6. (1 AND 2 AND 3 AND 4) NOT 5

Web of Science Core Collection, 755 Results

1. TS = (obes* OR adipos* OR “excess weight” OR overweight OR overnutrition OR fatness)

2. TS = (child* OR preschool* OR pupils OR schoolchild*)

3. TS = (diet OR dietar* OR food* OR eat* OR snack* OR sweets OR candy OR fastfood OR junkfood OR beverage* OR lemonade* OR “soda pop*” OR drink* OR softdrink*)

4. TS = (internet OR web* OR app OR apps OR campaign* OR “vending machine*” OR “automatic dispens*” OR canteen* OR school* NEAR/2 (cafe* OR “tuck shop*” OR lunchroom*) OR “product placement*” OR advergame* OR game* OR toy* OR plaything* OR advert* OR promotion* OR “public relations” OR marketing OR sponsor* OR brand* OR commercials OR packag* OR “product design” OR “portion size*” OR “large* portion*” OR “large* food portion*” OR “in store”)

5. TS = “health promotion*”

6. (1 AND 2 AND 3 AND 4) NOT 5

The Cochrane Library, 8 Results

1. [mh overnutrition] OR [mh overweight] OR (obes* OR adipos* OR “excess weight” OR overweight OR overnutrition OR fatness):ti,ab,kw

2. [mh child] OR [mh “child, preschool”] OR (child* OR preschool* OR pupils OR schoolchild*):ti,ab,kw

3. [mh “health knowledge, attitudes, practice”] OR [mh ^diet] OR [mh “food and beverages”] OR [mh “food industry”] OR [mh“food habits”] OR [mh ^“child nutritional physiological phenomena”] OR [mh eating] OR [mh ^“drinking behavior”] OR (diet OR dietar* OR food* OR eat* OR snack* OR sweets OR candy OR fastfood OR junkfood OR beverage* OR lemonade* OR “soda pop*” OR drink* OR softdrink*):ti,ab,kw

4. [mh internet] OR [mh “play and playthings”] OR [mh “persuasive communication”] OR [mh “advertising as topic”] OR [mh ^“public relations”] OR [mh marketing] OR [mh “product packaging”] OR [mh “portion size”] OR (internet OR web* OR app OR apps OR campaign* OR “vending machine*” OR “automatic dispens*” OR canteen* OR school* NEAR/3 (cafe* OR “tuck shop*” OR lunchroom*) OR “product placement*” OR advergame* OR game* OR toy* OR plaything* OR advert* OR promotion* OR “public relations” OR marketing OR sponsor* OR brand* OR commercials OR packag* OR “product design” OR “portion size*” OR “large* portion*” OR “large* food portion*“OR “in store”):ti,ab,kw

5. [mh“health promotion”] OR “health promotion*”:ti,ab,kw

6. (1 AND 2 AND 3 AND 4) NOT 5

PsycINFO, PSYNDEX, EconLit, Business Source Premier, 298 Results

1. with DE = Thesaurus terms from on the one hand PsycINFO/PSYNDEX and on the other hand EconLit/BSP

2. with AG = Age Group

3. with TI (Title), AB (Abstract) and KW (Author-Supplied Keywords) in sequence and additionally in German

4. with school* N2 (cafe* OR “tuck shop*” OR lunchroom*)

DE + TI/AB/KW in German + AG:

1. (DE (“Overweight” OR “Obesity” OR “Obesity (Attitudes Toward)”) OR

TI/AB/KW (übergewicht* OR “adipositas” OR adipös* OR fettleibig*))

2. (AG (“Childhood” OR “Preschool Age” OR "School Age”) OR DE (“CHILD consumers”) OR

TI/AB/KW (kind* OR schüler* OR schulkind* OR grundsch*))

3. (DE (“Health Knowledge” OR “Food” OR “Food Intake” OR “Eating Behavior” OR “Nutrition” OR “Beverages (Nonalcoholic)” OR “Drinking Behavior”) OR

DE (“FOOD industry” OR “BEVERAGE industry” OR “CANDY industry” OR “SNACK food industry” OR “SOFT drink industry suppliers” OR “SOFT drink industry”) OR

TI/AB/KW (nahrung* OR ernährung* OR ess* OR trink* OR getränk* OR limonade* OR “imbiss” OR süß*))

4. (DE (“Advertising” OR “Marketing” OR “Social Marketing” OR “Public Relations” OR “Product Design” OR “Brand Preferences” OR "Brand Names” OR “Internet” OR “Websites” OR “Television Advertising” OR “Toys” OR “Games” OR “Computer Games”) OR DE (“ADVERTISING & children” OR “INTERNET advertising & children” OR “GIFTS for children” OR “FOOD—Packaging” OR “ADVERTISING—Fast food restaurants” OR “FAST food restaurants—Public relations” OR “TELEVISION commercials” OR “TELEVISION advertising” OR “ADVERTISING” OR “ADVERTISING in motion pictures” OR “ADVERTISING—Motion pictures” OR “MARKETING” OR “viral marketing” OR “CORPORATE sponsorship” OR “INTERNET marketing” OR “MARKETING channels” OR “SOCIAL marketing” OR “APPLICATION software” OR “INTERNET” OR “WORLD Wide Web” OR “WEBSITES” OR “INTERNET television” OR “INTERNET advertising” OR “INTERNET pop-up advertising” OR “PUBLIC relations” OR "PROMOTIONAL television programs" OR “AUTOMATIC vending machines” OR “SCHOOL lunchrooms, cafeterias, *etc*.—Management” OR “BRAND name products” OR “BRAND image” OR “ADVERTISING—Brand name products” OR “PRODUCT design” OR “POINT-of-sale advertising” OR “PRODUCT placement” OR “PRODUCT placement in mass media” OR “IN-game advertising (Electronic games)” OR “ADVERTISING—Computer games” OR “VIDEO games—Advertising”) OR

TI/AB/KW (internetmarketing* OR internetwerb* OR “internetpromotion” OR onlinemarketing* OR onlinewerb* OR kampagne* OR automat* OR getränkeautomat* OR snackautomat* OR kantine* OR schulkantine* OR schulcafe* OR schulimbiss* OR schule* N2 (cafe* OR imbiss*) OR spiel* OR computerspiel* OR werb* OR marketing* OR fernsehwerb* OR anzeige* OR “öffentlichkeitsarbeit” OR marke* OR verpack* OR produkt* OR portion*))

5. (DE (“health promotion” OR “gesundheitsförderung”) OR TI/AB/KW (“gesundheitsförderung”)

6. (1 AND 2 AND 3 AND 4) NOT 5

WISO Wissenschaften, 18 Results

((übergewicht* OR “adipositas” OR adipös* OR fettleibig*) AND

(kind* OR schüler* OR schulkind* OR grundsch*) AND

(nahrung* OR ernährung* OR “essen” OR essverhalten* OR essgewohnheit* OR snack* OR “fastfood” OR “fast food” OR “junkfood” OR “junk food” OR trink* OR getränk* OR limonade* OR softdrink* OR “imbiss” OR süß*) AND

(kampagne* OR automat* OR getränkeautomat* OR snackautomat* OR kantine* OR schulkantine* OR schulcafe* OR schulimbiss* OR schule* NDJ2 (cafe* OR imbiss*) OR “product placement*” OR *promotion OR “public relations” OR *marketing OR “öffentlichkeitsarbeit” OR *werb* OR *anzeige* OR sponsor* OR marke* OR verpack* OR produkt* OR spielzeug* OR “spiele” OR computerspiel* OR portion*))

NOT “gesundheitsförderung”

MEDPILOT, 5 Results

((übergewicht* OR “adipositas” OR adipös* OR fettleibig*) AND

(kind* OR schüler* OR schulkind* OR grundsch*) AND

(nahrung* OR ernährung* OR ess* OR snack* OR “fastfood” OR “fast food” OR junkfood OR “junk food” OR trink* OR getränk* OR limonade* OR softdrink* OR “imbiss” OR süß*) AND

(internetmarketing* OR internetwerb* OR “internetpromotion” OR onlinemarketing* OR onlinewerb* OR kampagne* OR automat* OR getränkeautomat* OR snackautomat* OR kantine* OR schulkantine* OR schulcafe* OR schulimbiss* OR “product placement*” OR “promotion” OR "public relations” OR “marketing” OR “öffentlichkeitsarbeit” OR werb* OR fernsehwerb* OR anzeige* OR sponsor* OR marke* OR verpack* OR produkt* OR spielzeug* OR “spiele” OR computerspiel*
OR portion*))


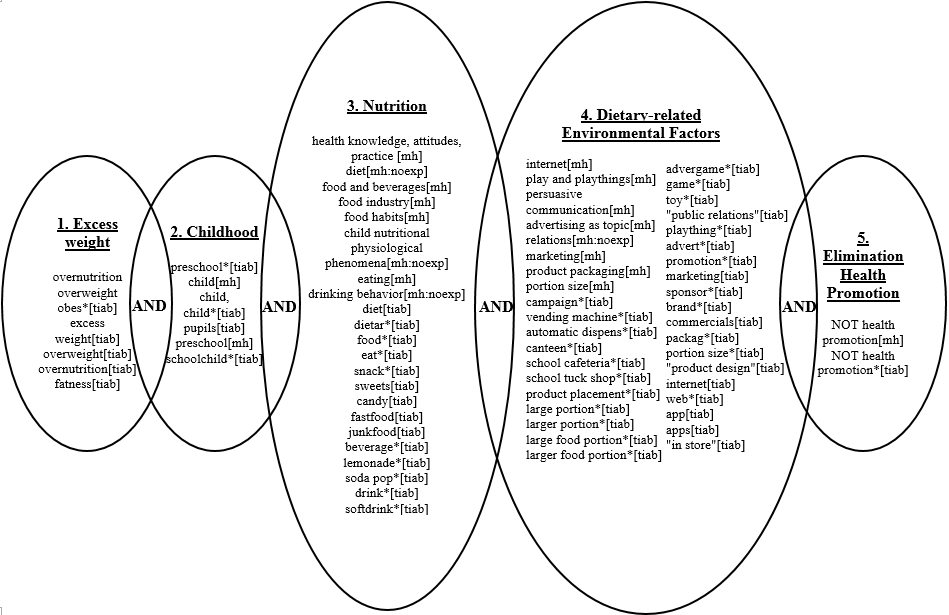


**Figure S2. A**ll Keywords in the concepts are connected with the OR-operator (was removed for practicability reasons).

**Table S1.** General characteristics of included studies.

| **Author** | **Year** | **Title** | **Country** | **Quanti-Tative Study Designs *** | **Target Group Age **** | **Sample Size** |
| --- | --- | --- | --- | --- | --- | --- |
| ***0 = does not apply, 1 = descriptive, 2 = content, 3 = correlational, 4 = quasi-experiment, 5 = experiment, I = Cross-sectional, II = Longitudinal** | | | | | | |
| **** = age not reported** | | | | | | |
| Briefel *et al*. | 2009 | School Food Environments and Practices Affect Dietary  Behavior of US Public School Children | USA | 1, I | 2 to 15 | Elem. school 732 children; Middle school 787 children |
| Adams *et al*. | 2012 | Effect of Restrictions on Television Food Advertising to Children on Exposure to Advertisements for Less Healthy' Foods: Repeat Cross-Sectional Study | UK | 2, I | 4 to 15 | 1.672.417 advertising person-minute-views |
| Andreyeva *et al.* | 2011 | Exposure to Food Advertising on Television: Associations with  Children’s Fast Food and Soft Drink Consumption and Obesity | USA | 4, II | 5 to 14 | 9760 children |
| Beales, Kulick | 2013 | Does Advertising on Television Cause Childhood Obesity?  A Longitudinal Analysis | USA | 4, II | 0 to 12 | 1868 children |
| Bell *et al.* | 2009 | Frequency and Types of Foods Advertised on Saturday Morning and Weekday Afternoon English- and  Spanish-Language American Television Programs | USA | 2, I | 0 | 7691 television advertise-ments |
| Berry, McMullen | 2008 | Visual Communication to Children in the Supermarket  Context: Health Protective or Exploitive? | Canada | 2, I | 2 to 8 | 2755 cereal boxes,  15 supermarkets |
| Buijzen *et al*. | 2008 | Associations between Children's Television Advertising Exposure and Their Food Consumption Patterns:  A Household Diary-Survey Study | Netherlands | 4, I | 4 to 12 | 234 parents |
| Carter *et al*. | 2011 | Children's Understanding of the Selling Versus Persuasive Intent of Junk Food Advertising: Implications for Regulation | Australia | 5, I | 4 to 12 | 594 children |
| Chernin | 2008 | The Effects of Food Marketing on Children's Preferences:  Testing the Moderating Roles of Age and Gender | USA | 5, I | 5 to 11 | 133 children |
| Cicchirillo, Lin | 2011 | Stop Playing with Your Food. A Comparison of For-Profit and Non-Profit Food-Related Advergames | USA | 2, I | 0 | 80 advergames |

**Table S1.** *Cont.*

| **Author** | **Year** | **Title** | **Country** | **Quanti-Tative Study Designs *** | **Target Group Age **** | **Sample Size** |
| --- | --- | --- | --- | --- | --- | --- |
| ***0 = does not apply, 1 = descriptive, 2 = content, 3 = correlational, 4 = quasi-experiment, 5 = experiment, I = Cross-sectional, II = Longitudinal** | | | | | | |
| **** = age not reported** | | | | | | |
| Dixon *et al*. | 2007 | The Effects of Television Advertisements for Junk Food Versus Nutritious Food on Children's Food Attitudes and Preferences | Australia | 5, I | 10 to 12 | 858 |
| Dovey | 2011 | Responsiveness to healthy television (TV) food advertisements/commercials is only evident in children  under the age of seven with low food neophobia | UK | 5, I | 5 to 7 | 66 |
| Fleming-Milici *et al.* | 2013 | Amount of Hispanic Youth Exposure to Food and Beverage  Advertising on Spanish- and English-Language Television | USA | 2, I | 2 to 5, 6 to 11 | n/a |
| Forman *et al.* | 2009 | Food Branding Influences *Ad Libitum* Intake Differently in  Children Depending on Weight Status. Results of a Pilot Study | USA | 5, I | 4 to 6 | 43 children |
| Grier *et al.* | 2007 | Fast-Food Marketing and Children's Fast-Food Consumption: Exploring Parents’ Influences in an Ethnically Diverse Sample | USA | 4, I | 2 to 12 | 312 children |
| Harris *et al.* | 2009 | Priming Effects of Television Food  Advertising on Eating Behavior | USA | 5, I | 7 to 11 | 108 |
| Huang, Yang | 2013 | Buy what is advertised on Television ? Evidence from  bans on child-directed food advertising. | USA | 4, I | 2 to 11,  12 and up | 13985 households |
| Jones | 2007 | Gross for Kids but Good for Parents: Differing Messages  in Advertisements for the Same Products | Australia | 3, I | 0 | 100 adults |
| Jones, Kervin | 2011 | An Experimental Study on the Effects of Exposure to  Magazine Advertising on Children's Food Choice | Australia | 5, I | 5 to 12 | 47 children |
| Keller *et al.* | 2012 | The Impact of Food Branding on Children's  Eating Behavior and Obesity | USA | 5, I | 4 to 6, 7 to 9 | 43 children/ 41 children/ 16 children |

**Table S1.** *Cont.*

| **Author** | **Year** | **Title** | **Country** | **Quanti-Tative Study Designs *** | **Target Group Age **** | **Sample Size** |
| --- | --- | --- | --- | --- | --- | --- |
| ***0 = does not apply, 1 = descriptive, 2 = content, 3 = correlational, 4 = quasi-experiment, 5 = experiment, I = Cross-sectional, II = Longitudinal** | | | | | | |
| **** = age not reported** | | | | | | |
| Kopelman *et al.* | 2007 | Advertising of Food to Children: Is Brand  Logo Recognition Related to their Food  Knowledge, Eating Behaviours and Food Preferences? | UK | 1, I | 9 to 11 | 476 children |
| Lascu *et al.* | 2013 | Online Marketing of Food Products to Children: The Effects of National Consumer Policies in High-Income Countries | France, Spain, USA | 2, I | 0 | 58 US, 40 French, 63 Spanishwebsites |
| Manganello *et al.* | 2013 | A Content Analysis of Food Advertisements Appearing  in Parenting Magazines | USA | 2, I | 0 | 476 adverts |
| McAlister, Cornwell | 2012 | Collectible Toys as Marketing Tools: Understanding Preschool Children's Responses to Food Paired with Premiums | USA | 5, I | 3 to 5 | 85 children/ 56 children |
| Ogba, Johnson | 2010 | How Packaging Affects the Product Preferences of Children and the Buyer Behaviour of their Parents in the Food Industry | UK | 1, I | 0–3, 4-6,  7–10, 11–13, 14+ | 145 parents |
| Osei-Assibey *et al.* | 2012 | The Influence of the Food Environment on Overweight and  Obesity in Young Children: A Systematic Review | USA,  Netherlands, UK,  Germany,  Sweden | 0 | 2 to 13 | 35 studies |
| Paek | 2014 | Characteristics of Food Advergames that reach Children and the Nutrient Quality of the Foods they Advertise | USA | 3, I | 2 to 11 | 44 advergames  reaching children;  99 not reaching them |
| Page | 2009 | Emotional and rational product appeals in televised food advertisements for children: analysis of commercials  shown on US broadcast networks | USA | 2, I | 0 | 147 |

**Table S1.** *Cont.*

| **Author** | **Year** | **Title** | **Country** | **Quanti-Tative Study Designs *** | **Target Group Age **** | **Sample Size** |
| --- | --- | --- | --- | --- | --- | --- |
| ***0 = does not apply, 1 = descriptive, 2 = content, 3 = correlational, 4 = quasi-experiment, 5 = experiment, I = Cross-sectional, II = Longitudinal** | | | | | | |
| **** = age not reported** | | | | | | |
| Potvin Kent | 2011a | Food marketing on children's television in  two different policy environments | Canada | 3, I | 10 to 12 | 428 |
| Potvin Kent | 2012 | A Nutritional Comparison of Foods and Beverages Marketed to Children in Two Advertising Policy Environments | Canada | 3, I | 10 to 12 | 428 |
| Potvin Kent | 2014 | The influence of the Children's Food and Beverage  Advertising Initative: change in children’s exposure to food advertising on television in Canada between 2006–2009 | Canada | 3, I | 2 to 11 | Toronto: 167 ('06), 188 ('09) and 242 ('11) / Vancouver: 78 ('06), 83 ('09) and 142 ('11) |
| Potvin Kent | 2011b | Self-regulation by industry of food marketing is having  little impact during children’s preferred television | Canada | 3, I | 10 to 12 | 272 |
| Schwartz *et al.* | 2010 | Breakfast Cereal Industry Pledges to Self-Regulate Advertising  to Youth: Will They Improve the Marketing Landscape? | USA | 3, I | 2 to 11 | 83 cereal brands |
| Warren *et al.* | 2007 | Food and Beverage Advertising to Children on US  Television: Did National Food Advertisers Respond | USA | 2, II | 0 | commercials: 1880 in 2004/05 and 2898 in 2006 |
| Wicks *et al.* | 2009 | Dual-modality disclaimers, emotional appeals and  production techniques in food advertising airing  during programs rated for children | USA | 2, I | 2 to 11 | 3893 |
| Yu | 2012 | Mother's Perceptions of the Negative Impact on  TV Food Ads on Children's Food Choices | USA | 1, I | 7 to 12 | 318 mothers |

**Table S2.** Key obesogenic environments influenced by the food industry found to promote childhood overweight and obesity.

| **Author** | **A** | **B** | **C** | **Key Findings** |
| --- | --- | --- | --- | --- |
| A: Marketing Technique | 1 = advertisement, 2 = product placement, 3 = celebrities, 4 = canteen, 5 = vending machine, 6 = advergames, 7 = websites, 8 = viral marketing, 9 = sponsorship, 10 = packaging, 11 = portion sizes, 12 = in-store placement, 13 = personification, 14 = brand image | | | |
| B: Influence of industry found | 0 = no, 1 = yes, 2 = does not apply | | | |
| C: Impact on | 1 = preferences, 2 = behavior, 3 = BMI, 4 = parents, 5 = does not apply | | | |
| **1. School** | | | | |
| Briefel *et al*. | 4, 5, 9, 14 | 1 | 2 | The relationship between school health policies/food environments and children’s food consumption in kilocalories was measured in a nationally representative sample of public schools. The items concerning the influence of the industry were “no pouring rights contract” (43.1% ± 6.3 of all elementary schools, 35.4% ± 6.7 of middle schools), “no items offered from brand-name restaurants” (66.9% ± 6.3, 65.0% ± 6.6) and “no vending machines” (75.6% ± 5.4, 8.5% ± 3.3) with an additional item called "has vending, but no low-nutrient, energy-dense foods or beverages” (17.1% ± 5.1, 18.8% ± 4.6). Children in elementary school report eating lunch and snacking more often in school than middle school children (97.1% ± 0.79 *vs*. 91.2% ± 1.62; 44.6% ± 3.31 *vs.* 29.2% ± 2.8). |
| **2. Home** | | | | |
| Grier *et al*. | 1 | 1 | 2, 4 | The authors found that parent’s reported exposure to fast-food promotion was directly associated with children’s  consumption of fast food (χ^2^ = 5.23, *p* = 0.02). In addition, African American and Hispanic children were the most  likely to consume fast-food frequently while non-Hispanic white and children of mixed race ate fast-food the least often. |
| Jones | 1, 13, 14 | 1 | 4 | The strategy of purposely advertising a product differently to the parent than to the child has an  influence on the impact of the advert. With all products, except 'Whipped Cream', an adult was more than  double as likely to buy the product for a child if he/she had seen the adult version of an advertisement  (χ^2^ from 5.88 to 18.92) than if he had seen an advertisement for the same product directed towards children.  Furthermore, on average, they believed that the product was more healthy, nutritionally beneficial and  convenient in this case. If they had seen the child version, they thought that it was more fun, exciting and popular. |

**Table S2.** *Cont.*

| **Author** | **A** | **B** | **C** | **Key Findings** |
| --- | --- | --- | --- | --- |
| Manganello *et al*. | 1, 3, 14 | 2 | 5 | The foods most commonly advertised to parents in magazines were snacks (13%), followed by baked goods, meat and fruit juice. The message most often used to sell the product was taste (55%) in addition to newness (24%) and convenience (17%). Only one percent of the adverts included the argument that the product was economical. Eight percent focused on a celebrity. Concerning the nutritiousness of products, 22% of the adverts claimed that the product was natural, and 18% that it would improve one's health and/or improve physical/mental performance (26%). |
| Yu | 1, 8 | 1 | 1, 2, 4 | The mothers' answers to a survey including different statements about children and TV food suggested  that TV food adverts encourage unhealthy eating habits and lead to nagging behavior (57.1% agreed) ,  *i.e.*, the children's preferences were influenced (77.92% agreed) and the adverts tricked the children (59.31%). They did not believe that food adverts were the most important influence on their children's eating habits (only 7.57% agreed). |
| **3. Internet** | | | | |
| Cicchirillo, Lin | 1, 6, 13, 14 | 2 | 5 | The hypotheses that for-profit games focus on product-related messages more (χ^2^ = 30.35, *p* < 0.01) and non-profit advergames on health-related messages (χ^2^ = 31.75, *p* < 0.01) were supported. Additionally, for-profit advergames presented the company’s identifiers more often (χ^2^ = 18.34, *p* < 0.01) and were more likely to include sponsored messages (χ^2^ = 15.31, *p* < 0.01). |
| Lascu *et al.* | 1, 3, 6, 7, 8, 10, 14 | 2 | 5 | No significant differences were found between US-, French and Spanish food company websites concerning the use of marketing features. There was a trend of US-American websites being more rewards- and brand-oriented than French and Spanish websites. French websites focused less on games but more on interactions and Spanish websites included less nutritional information. Overall, the feature category of which a very little number of features was used was “rewards” and those which were used most often were “nutrition” and “games.” |
| Paek | 1, 6 | 2 | 5 | The only significant (*p* < 0.01) predictors of an advergame reaching a child were if no ad breaks were present  (β = 2.39 ± 0.77) and the number of brand identifiers (β = 0.71 ± 0.27). A trend could be seen, showing that the advergames reaching children were less healthy by the definition of the IOM and the CSPI guidelines but not by the FDA guidelines. An additional information collected by this study was that 11.4% of the advergames reaching children in fact included an age limit, which did not allow children to access this site in theory. |
| **4. Television** | | | | |
| Adams *et al.* | 1 | 2 | 5 | 13.3% of all TV adverts directed at 4 to 15-year old children promoted food. Of these, 49.8% were for HFSS (high fat, salt or sugar) food. The share of adverts containing HFSS food of all person-minute views of children was 6.6%. |

**Table S2.** *Cont.*

| **Author** | **A** | **B** | **C** | **Key Findings** |
| --- | --- | --- | --- | --- |
| Andreyeva *et al.* | 1 | 1 | 2, 3 | An increase of about 33 adverts for sugar-sweetened carbonated soft drinks per year watched by children was accompanied by a 9.4% increase in the consumption of soft drinks (*p* = 0.01). A similar increase of fast food advertisement does not significantly increase consumption of fast food. It did increase by 7.4% though (*p* = 0.01), when carbonated soft drinks were advertised more. Overall, changes in the amount of food advertisement did not have a significant impact on BMI z-scores. Amongst children with BMI ≥ 85th percentile, an increase of 1.5% from a mean BMI z-score was found. |
| Beales, Kulick | 1 | 0 | 3 | For the younger sample aging from 0 to 7 years, none of the examined television variables had a significant effect on BMI and it was shown that BMI was influenced by baseline BMI, age and parents (their BMI, cleanliness and amount of sleep time). For the older sample aging from 7 to 12 years, total television viewing time was significant (*p* = 0.05), but rather current than baseline television viewing, which implies that the viewing time did not have a causal effect on BMI. There was no significant difference between non-commercial and commercial TV viewing time. |
| Bell *et al*. | 1 | 2 | 5 | This content analysis found that 31.7% of all adverts on children’s television programs were food adverts and thus significantly more numerous than on other channels (*p* = 0.001). The types of foods advertised predominantly had high fat or sugar content (70.5%). Adverts for fruits, vegetables and juices, of which 73.7% were for juices, made up only 1.7% of all food adverts. Compared to adult channels, children’s channels showed significantly more adverts for cereals (30.9% to 15.5%) and sweets (20.6% to 13.6%; *p* = 0.05), but significantly less for fast food (13.6% to 28.4%). |
| Buijzen *et al.* | 1, 14 | 1 | 2 | Exposure to TV food advertising was related to the food brand choice of children (β = 0.21, *p* < 0.01) (children chose more foods of the advertised brands) and the consumption of energy-dense foods in general (β = 0.19; *p* < 0.01). Overall food intake was only significantly influenced in children of low-income families (β = 0.12, *p* < 0.05). |
| Chernin | 1 | 1 | 1 | The hypothesis that the preference for two products is positively correlated with watching their commercials was supported. There was no significant difference in preference for one of the products according to gender or age of the participants. Old and young children were equally persuaded by the commercials, whereas boys were influenced more than girls. |

**Table S2.** *Cont.*

| **Author** | **A** | **B** | **C** | **Key Findings** |
| --- | --- | --- | --- | --- |
| Dixon *et al.* | 1 | 1 | 1 | This cross-sectional study found that the exposure to TV was positively correlated with consumption and positive attitudes about fast food. There was an increased preference of healthy foods among children exposed to healthy food adverts compared to non-exposed children, but no increased preference of junk food among children exposed to unhealthy food adverts. Airing junk food adverts together with healthy food adverts did not reduce the impact of junk food adverts, but had a negative impact on attitudes towards vegetables. Children who watched healthy food adverts also did not have higher nutrition knowledge. |
| Dovey | 1 | 1 | 2 | The study revealed that the total food intake of children in kcal was higher after watching unhealthy food  adverts compared to adverts for healthy food (*p* = 0.005) or toys (*p* < 0.001). Children with high food  neophobia ate more chocolate after watching both unhealthy (*p* = 0.045) and healthy (*p* = 0.036) food adverts.  Those with low food neophobia ate less chocolate after watching healthy food adverts compared to unhealthy  adverts only (*p* = 0.003). Weight status did not affect the food intake after food adverts significantly. |
| Fleming-Milici *et al*. | 1 | 2 | 5 | There is a difference between the amount of adverts viewed per hour in English-language television of 2 to 5-year old children (3.2 adverts) and 6 to 11-year old children (3.7 adverts) for Hispanic children and also for non-Hispanic youth (3.6 adverts *vs.* 4.0 adverts). English-language television included significantly more breakfast cereal, prepared foods and meal, snack food and fruit juice and non-carbonated beverage adverts than Spanish-language TV (*p* < 0.01). Spanish-language TV had significantly more adverts for fast food restaurants and carbonated regular beverages (*p* < 0.01). |
| Harris *et al*. | 1 | 1 | 2 | The prediction that children eat more when watching television including food advertisement, opposed to when watching television with non-food advertisement added, was confirmed. Children in the food advertisement condition ate significantly more snacks in 14 min while watching than children in the control group (mean intake of 19.7 gr. of goldfish crackers *vs.* 28.5 gr.; *p* = 0.01). If children watched television with food adverts just for 30 minutes a day and ate crackers at this rate, they would consume an additional 94 kcal. than if there were no food adverts. |
| Huang, Yang | 1 | 2 | 5 | Companies in the Children's Food and Beverage Advertising Initiative (CFBAI) pledged to devote 50 % of  their adverts to healthier products and not to direct adverts to children under 12 years. One of the examined companies entirely stopped their advertising for the product examined. As a consequence, the exposure to this brand's adverts of children from age 2 to 11 was reduced greatly (*p* < 0.01(two-tailed)). The relative purchasing frequency of this product per household was reduced by 80% on average (*p* < 0.01). The authors conclude that the CFBAI’s success is limited when companies advertise to a more general audience not limited to children below 12 years of age. |

**Table S2.** *Cont.*

| **Author** | **A** | **B** | **C** | **Key Findings** |
| --- | --- | --- | --- | --- |
| Page | 1 | 2 | 5 | Of the emotional appeals used in TV food commercials, “fun/happiness” was used most often (85%). “Play” (58.5%), “fantasy/imagination” (50.3%) and “social enhancement/peer acceptance” (34%) were also common. The only rational appeal used in more than half of the adverts was “taste/flavor” (52.4%). Mentioning of the healthiness/nutritiousness of the product was very rare (8.2%) and the value for money-aspect never appeared. 97.3% of the examined commercials had a story format. |
| Potvin Kent | 1 | 2 | 5 | The effect of a children’s TV advertising ban in Canada was examined. The ban did not reduce the  number of food and beverage adverts in total. The amount of beverage adverts increased (*p* < 0.01).  The amount of food adverts (*p* < 0.001), the use of fun as persuasive appeal (*p* < 0.001) and the use of  media characters or celebrities (*p* < 0.04) decreased in comparison to a non-effected region. There was a  significantly lower frequency of candy and snack promotions in the affected region (*p* < 0.001). |
| Potvin Kent | 1 | 2 | 5 | Contrary to expectations, the companies participating in the CFBAI were responsible for significantly more food and beverage promotions during children’s total preferred viewing (*p* = 0.009) and during their preferred viewing on children’s specialty channels (*p* = 0.001). Furthermore, the CFBAI group used significantly more media characters to attract children. Their products were higher in fat, sugar, energy and sodium per 100g (*p* < 0.001). In total 80 % of the CFBAI food and beverage commercials promote less healthy products compared to 55 % among the non-CFBAI products (*p* = 0.001). |
| Potvin Kent | 1 | 2 | 5 | The effects of the CFBAI (self-regulation) were examined by comparing the region where it applied to another region of Canada. The hypothesis that children affected by the initiatives are exposed to food adverts that are lower in sugar and more healthful could be supported. The affected adverts were significantly lower in sugar per 100 g (*p* < 0.05) and in average sugar as percentage of energy (*p* < 0.05). In the non-affected groups there were significantly more foods and beverages considered as „less healthy“ (*p* < 0.001). |
| Potvin Kent | 1 | 2 | 5 | It was hypothesized that after implementation of the CFBAI a reduction in food and beverage advertising  and in children’s exposure would be observed. There was a reduction of 4.5 % in the number of food and beverage  adverts on children’s specialty channels. Contrary to expectations, it was found that children’s total average exposure has increased by 5.4%/ 2.5% in two intervention cities. This was mainly due to a large increase in exposure to adverts for the snack category. |

**Table S2.** *Cont.*

| **Author** | **A** | **B** | **C** | **Key Findings** |
| --- | --- | --- | --- | --- |
| Schwartz *et al*. | 1 | 2 | 5 | Cereal advertising comprised 17% of all TV food adverts seen by children, while the average child was exposed to approximately 758 cereal adverts in 2007. This is 136% higher than the combined adults’ and adolescents’ and 30% more than adults’ cereal advertising exposure. Unhealthy cereals made up 98% of the cereal advertising exposure for children and children’s cereals had worse nutrition than non-children-cereals (*p* < 0.0001). The 2–11 year old children’s exposure to cereal advertising was significantly related to the nutrition score (higher score implies “less healthy”). |
| Warren *et al*. | 1 | 2 | 5 | There was no major change in types of food products, persuasive appeals and production techniques in commercials, after the introduction of a new policy (in 2005), in which major food companies pledged to stop targeting children under 12 with adverts for unhealthy food. Pizza and fast food were still the most frequently advertised products concerning child-targeted adverts and in child-rated shows. The categories “taste, flavour, smell” and “mood alteration” remained the most frequently used persuasive appeals. Of the production techniques, visual effects were still the most seen, but there was a 10% increase in animations as production technique among the child-targeted commercials. |
| Wicks *et al*. | 1 | 2 | 5 | Food adverts appearing in children’s programming use significantly more of production techniques like animation and jingles than food adverts directed to adults. The same holds for emotional appeals resulting in changing moods if children watch food adverts e.g., more happiness. Most disclaimers in child-related programs were not dual modality (information is presented in audio and video) and thus, not easy to understand for children. |
| **5. Promotional Campaigns** | | | | |
| Carter *et al*. | 1 | 1 | 1 | Only 38% of the 7 to 8 year old children were able to identify a McDonald ad’s true intent by showing the right picture, but already 64% of them could express this verbally (compared to 28% at age 6 to 7). The age at which the persuasive intent could also be expressed nonverbally by the majority of the children is 8 to 9 (57%). Both nonverbal and verbal ability to convey the knowledge of this intent increased until over 90% at age 8 to 9. |

**Table S2.** *Cont.*

| **Author** | **A** | **B** | **C** | **Key Findings** |
| --- | --- | --- | --- | --- |
| Forman *et al*. | 1, 10, 14, 11 | 0 | 2 | Food brand knowledge did not differ between overweight and non-overweight children. The awareness of brands of unhealthy foods was higher than for healthy foods, and brand awareness increased with child age (*r* = 0.6;  *p* ≤ 0.001). Overall, no difference was found between the amount consumed in two conditions with either branded or unbranded food. However, the difference was significant between overweight (41 kcal more when branded) and non-overweight children (45 kcal less when branded; *p* ≤ 0.05). |
| Jones, Kervin | 1 | 1 | 1, 2 | Those children who read a magazine including adverts chose more of the advertised food items when presented  with a variety of healthy and unhealthy (advertised and non-advertised) snacks (mean of 1.64 out of 2 compared with 1.32; *p* = 0.046) than those reading a magazine without adverts. Interestingly, when questioned beforehand about the factors for choosing a snack food, 86% named taste and 87.2% named health as important factors. |
| Keller *et al*. | 1, 10, 14, 11 | 1 | 2, 3 | Based on a study reported in the article by Forman *et al*. (2009), a color stroop task was compared to a food brand stroop task. Overweight children took longer than non-overweight children to respond when the image was not congruent to the writing. There was a trend of children eating more food if it was branded than if it was unbranded (*p* = 0.07), which did not differ between overweight and non-overweight children. Girls ate significantly more in the branded than in the unbranded condition (*p* < 0.05). In another study, there was an increased vegetable intake when children received gifts alongside the vegetables (*p* < 0.05) and the BMI z-score decreased while the control group children's z-score increased (*p* < 0.05). |
| Kopelman *et al*. | 14 | 0 | 1, 2 | First of all, this study reports that children in the UK have a poor diet, shown by 72.1% of them consuming crisps, 70.9% sweets and 60.2% soda drinks at least once a day. On the other hand, 56.9% ate five or more portions of fruits and vegetables per day. The consumption of fried food was significantly more likely in children of low  socio-economic status and with unemployed parents (χ^2^ = 27.5; χ^2^ = 41.1). A high brand logo recognition by the children of 88% was shown. This did not significantly correlate with eating behavior, food knowledge or preferences. Concerning food knowledge, boys and children of unemployed parents were more likely to have poor food knowledge (χ^2^ = 13.1; χ^2^ = 11.3). |

**Table S2.** *Cont.*

| **Author** | **A** | **B** | **C** | **Key Findings** |
| --- | --- | --- | --- | --- |
| McAlister, Cornwell | 1, 10, 14 | 1 | 1, 2 | The hypothesis that collectible toys make a menu more attractive than noncollectible toys (F = 41.42, *p* < 0.001) was confirmed. This effect was stronger for a healthful than for a less healthful menu (F = 14.19, *p* < 0.001, partial η^2^ = 0.56). Although children preferred a less healthful menu to a more healthful one if there was no toy or a noncollectible toy added (F = 96.16, *p* < 0.001), they did not evaluate the menus differently if there was a collectible toy added to both (F = 2.18, n.s.). If a collectible toy was added which the child already owned, the value of the menu reported by the child was similar to the condition in which the toy was noncollectible. |
| **6. Food Retailers** | | | | |
| Berry, McMullen | 3, 10, 12 | 2 | 5 | One third of the products which were in a child’s visual field of view in a supermarket had boxes with a spokes-character and one third had a child incentive on them. Almost 90% of the products had packaging in highly or moderately child-oriented colors. On average a child was able to reach 75% of the products that it could see. If a box displayed a spokes-character, had child-themed shapes or colors, incentives or in an upper shelf, it contained more sugar (*p* < 0.05). |
| Ogba, Johnson | 3, 10, 12, 13 | 1 |  | Parents were asked to fill out an item questionnaire concerning the effects of food packaging on their children and themselves. All three hypotheses “Packaging has a strong impact on children’s product preferences,” “Children are more likely to influence the purchase of unhealthy foods due to influence from packaging” and “Children have a strong influence on their parent’s buyer behavior in food choices for children” were confirmed with Pearson Correlations between 0.65 and 0.87. |
| Osei-Assibey *et al.* | 1, 11, 14 | 1, 0 | 1, 2, 3, 4 | In general, the studies suggested that the food environment does have an influence on overweight in young children. Concerning food promotion, seven studies examined this and all found an effect. They stated that the advertised foods do not adhere to health guidelines. Five studies addressed the topic of portion sizes and all found an effect although some limited to boys or school-aged children. Six studies supported the hypothesis that  sugar-sweetened soft drinks increase the risk of being overweight. |

CFBAI: Children’s Food and Beverage Advertising Initiative; HFSS: High fat, salt or sugar; BMI: body-mass-index; p-value: indictor of significance level of statistical test; F-value: indicator of how much of the variance in data can be explained by hypothesis; χ2: indicator of goodness of fit of observed to theoretical distribution; β: how many standard deviations dependent variable will change, per standard deviation increase in predictor variable in linear regression model.

© 2015 by the authors; licensee MDPI, Basel, Switzerland. This article is an open access article distributed under the terms and conditions of the Creative Commons by Attribution (CC-BY) license (http://creativecommons.org/licenses/by/4.0/).
